# Supplementary material for: HIVprotI: an integrated web based platform for prediction and design of HIV proteins inhibitors
Source: J Cheminform. 2018 Mar 9;10:12. doi: 10.1186/s13321-018-0266-y (PMC5845081; doi:10.1186/s13321-018-0266-y)
Supplement: Supplementary file 2 — Additional file 2. Source code of HIVProtI web server. [file 13321_2018_266_MOESM2_ESM.zip › HIVprotI_Source-code/hivprotI/weka-3-6-6/doc/weka/core/xml/XMLInstances.html]

XMLInstances


---


|  |  |  |
| --- | --- | --- |
| **Overview** \| **Package** \| **Class** \| **Tree** \| **Deprecated** \| **Index** \| **Help** \| **Weka's home** | |  |
| **PREV CLASS**   **NEXT CLASS** | **FRAMES**    **NO FRAMES**     **All Classes** |  |
| SUMMARY: NESTED \| FIELD \| CONSTR \| METHOD | DETAIL: FIELD \| CONSTR \| METHOD |  |


---


## weka.core.xml Class XMLInstances

```
java.lang.Object
  weka.core.xml.XMLDocument
      weka.core.xml.XMLInstances
```

**All Implemented Interfaces:**: java.io.Serializable, RevisionHandler

---

``` public class XMLInstances extends XMLDocument implements java.io.Serializable ```

XML representation of the Instances class.

**Version:**
:   $Revision: 1.4 $

**Author:**
:   fracpete (fracpete at waikato dot ac dot nz)

**See Also:**: Serialized Form

---

| **Field Summary** | |
| --- | --- |
| `static java.lang.String` | `ATT_CLASS`             the class attribute |
| `static java.lang.String` | `ATT_FORMAT`             the format attribute (for date attributes) |
| `static java.lang.String` | `ATT_INDEX`             the index attribute |
| `static java.lang.String` | `ATT_MISSING`             the missing attribute |
| `static java.lang.String` | `ATT_TYPE`             the type attribute |
| `static java.lang.String` | `ATT_VERSION`             the version attribute |
| `static java.lang.String` | `ATT_WEIGHT`             the weight attribute |
| `static java.lang.String` | `DOCTYPE`             the DTD |
| `static java.lang.String` | `FILE_EXTENSION`             The filename extension that should be used for xrff files |
| `static java.lang.String` | `TAG_ATTRIBUTE`             the attribute element |
| `static java.lang.String` | `TAG_ATTRIBUTES`             the attributes element |
| `static java.lang.String` | `TAG_BODY`             the body element |
| `static java.lang.String` | `TAG_DATASET`             the root element |
| `static java.lang.String` | `TAG_HEADER`             the header element |
| `static java.lang.String` | `TAG_INSTANCE`             the instance element |
| `static java.lang.String` | `TAG_INSTANCES`             the data element |
| `static java.lang.String` | `TAG_LABEL`             the label element |
| `static java.lang.String` | `TAG_LABELS`             the labels element |
| `static java.lang.String` | `TAG_METADATA`             the meta-data element |
| `static java.lang.String` | `TAG_NOTES`             the notes element |
| `static java.lang.String` | `TAG_PROPERTY`             the property element |
| `static java.lang.String` | `TAG_VALUE`             the value element |
| `static java.lang.String` | `VAL_DATE`             the value for date |
| `static java.lang.String` | `VAL_NOMINAL`             the value for nominal |
| `static java.lang.String` | `VAL_NORMAL`             the value for normal |
| `static java.lang.String` | `VAL_NUMERIC`             the value for numeric |
| `static java.lang.String` | `VAL_RELATIONAL`             the value for relational |
| `static java.lang.String` | `VAL_SPARSE`             the value for sparse |
| `static java.lang.String` | `VAL_STRING`             the value for string |

**Fields inherited from class weka.core.xml.XMLDocument**

`ATT_NAME, DTD_ANY, DTD_AT_LEAST_ONE, DTD_ATTLIST, DTD_CDATA, DTD_DOCTYPE, DTD_ELEMENT, DTD_IMPLIED, DTD_OPTIONAL, DTD_PCDATA, DTD_REQUIRED, DTD_SEPARATOR, DTD_ZERO_OR_MORE, PI, VAL_NO, VAL_YES`


**Constructor Summary**

`XMLInstances()`
  
          the default constructor

`XMLInstances(Instances data)`
  
          generates the XML structure based on the given data

`XMLInstances(java.io.Reader reader)`
  
          generates the Instances directly from the reader containing the
XML data.


| **Method Summary** | |
| --- | --- |
| `Instances` | `getInstances()`             returns the current instances, either the ones that were set or the ones that were generated from the XML structure. |
| `java.lang.String` | `getRevision()`             Returns the revision string. |
| `static void` | `main(java.lang.String[] args)`             takes an XML document as first argument and then outputs the Instances statistics |
| `void` | `setInstances(Instances data)`             builds up the XML structure based on the given data |
| `void` | `setXML(java.io.Reader reader)`             reads the XML structure from the given reader |

**Methods inherited from class weka.core.xml.XMLDocument**

`clear, evalBoolean, evalDouble, evalString, findNodes, getBuilder, getChildTags, getChildTags, getContent, getDocType, getDocument, getFactory, getNode, getRootNode, getValidating, newDocument, print, read, read, read, read, setDocType, setDocument, setRootNode, setValidating, toString, write, write, write, write`

**Methods inherited from class java.lang.Object**

`equals, getClass, hashCode, notify, notifyAll, wait, wait, wait`

**Field Detail**

### FILE\_EXTENSION

```
public static java.lang.String FILE_EXTENSION
```

:   The filename extension that should be used for xrff files

---


### TAG\_DATASET

```
public static final java.lang.String TAG_DATASET
```

:   the root element

    **See Also:**: Constant Field Values

---


### TAG\_HEADER

```
public static final java.lang.String TAG_HEADER
```

:   the header element

    **See Also:**: Constant Field Values

---


### TAG\_BODY

```
public static final java.lang.String TAG_BODY
```

:   the body element

    **See Also:**: Constant Field Values

---


### TAG\_NOTES

```
public static final java.lang.String TAG_NOTES
```

:   the notes element

    **See Also:**: Constant Field Values

---


### TAG\_ATTRIBUTES

```
public static final java.lang.String TAG_ATTRIBUTES
```

:   the attributes element

    **See Also:**: Constant Field Values

---


### TAG\_ATTRIBUTE

```
public static final java.lang.String TAG_ATTRIBUTE
```

:   the attribute element

    **See Also:**: Constant Field Values

---


### TAG\_LABELS

```
public static final java.lang.String TAG_LABELS
```

:   the labels element

    **See Also:**: Constant Field Values

---


### TAG\_LABEL

```
public static final java.lang.String TAG_LABEL
```

:   the label element

    **See Also:**: Constant Field Values

---


### TAG\_METADATA

```
public static final java.lang.String TAG_METADATA
```

:   the meta-data element

    **See Also:**: Constant Field Values

---


### TAG\_PROPERTY

```
public static final java.lang.String TAG_PROPERTY
```

:   the property element

    **See Also:**: Constant Field Values

---


### TAG\_INSTANCES

```
public static final java.lang.String TAG_INSTANCES
```

:   the data element

    **See Also:**: Constant Field Values

---


### TAG\_INSTANCE

```
public static final java.lang.String TAG_INSTANCE
```

:   the instance element

    **See Also:**: Constant Field Values

---


### TAG\_VALUE

```
public static final java.lang.String TAG_VALUE
```

:   the value element

    **See Also:**: Constant Field Values

---


### ATT\_VERSION

```
public static final java.lang.String ATT_VERSION
```

:   the version attribute

    **See Also:**: Constant Field Values

---


### ATT\_TYPE

```
public static final java.lang.String ATT_TYPE
```

:   the type attribute

    **See Also:**: Constant Field Values

---


### ATT\_FORMAT

```
public static final java.lang.String ATT_FORMAT
```

:   the format attribute (for date attributes)

    **See Also:**: Constant Field Values

---


### ATT\_CLASS

```
public static final java.lang.String ATT_CLASS
```

:   the class attribute

    **See Also:**: Constant Field Values

---


### ATT\_INDEX

```
public static final java.lang.String ATT_INDEX
```

:   the index attribute

    **See Also:**: Constant Field Values

---


### ATT\_WEIGHT

```
public static final java.lang.String ATT_WEIGHT
```

:   the weight attribute

    **See Also:**: Constant Field Values

---


### ATT\_MISSING

```
public static final java.lang.String ATT_MISSING
```

:   the missing attribute

    **See Also:**: Constant Field Values

---


### VAL\_NUMERIC

```
public static final java.lang.String VAL_NUMERIC
```

:   the value for numeric

    **See Also:**: Constant Field Values

---


### VAL\_DATE

```
public static final java.lang.String VAL_DATE
```

:   the value for date

    **See Also:**: Constant Field Values

---


### VAL\_NOMINAL

```
public static final java.lang.String VAL_NOMINAL
```

:   the value for nominal

    **See Also:**: Constant Field Values

---


### VAL\_STRING

```
public static final java.lang.String VAL_STRING
```

:   the value for string

    **See Also:**: Constant Field Values

---


### VAL\_RELATIONAL

```
public static final java.lang.String VAL_RELATIONAL
```

:   the value for relational

    **See Also:**: Constant Field Values

---


### VAL\_NORMAL

```
public static final java.lang.String VAL_NORMAL
```

:   the value for normal

    **See Also:**: Constant Field Values

---


### VAL\_SPARSE

```
public static final java.lang.String VAL_SPARSE
```

:   the value for sparse

    **See Also:**: Constant Field Values

---


### DOCTYPE

```
public static final java.lang.String DOCTYPE
```

:   the DTD


**Constructor Detail**

### XMLInstances

```
public XMLInstances()
             throws java.lang.Exception
```

:   the default constructor

    **Throws:**: `java.lang.Exception` - if XML initialization fails

---


### XMLInstances

```
public XMLInstances(Instances data)
             throws java.lang.Exception
```

:   generates the XML structure based on the given data

    **Parameters:**: `data` - the data to build the XML structure from **Throws:**: `java.lang.Exception` - if initialization/generation fails

---


### XMLInstances

```
public XMLInstances(java.io.Reader reader)
             throws java.lang.Exception
```

:   generates the Instances directly from the reader containing the
    XML data.

    **Parameters:**: `reader` - the reader for the XML data **Throws:**: `java.lang.Exception` - if something goes wrong


**Method Detail**

### setInstances

```
public void setInstances(Instances data)
```

:   builds up the XML structure based on the given data

    :   **Parameters:**: `data` - data to generate the XML from

---


### getInstances

```
public Instances getInstances()
```

:   returns the current instances, either the ones that were set or the ones
    that were generated from the XML structure.

    :   **Returns:**: the current instances

---


### setXML

```
public void setXML(java.io.Reader reader)
            throws java.lang.Exception
```

:   reads the XML structure from the given reader

    :   **Parameters:**: `reader` - the reader to get the XML from **Throws:**: `java.lang.Exception` - if

---


### getRevision

```
public java.lang.String getRevision()
```

:   Returns the revision string.

    :   **Specified by:**: `getRevision` in interface `RevisionHandler` **Overrides:**: `getRevision` in class `XMLDocument`
    :   **Returns:**: the revision

---


### main

```
public static void main(java.lang.String[] args)
```

:   takes an XML document as first argument and then outputs the Instances
    statistics

    :   **Parameters:**: `args` - the commandline options


---


|  |  |  |
| --- | --- | --- |
| **Overview** \| **Package** \| **Class** \| **Tree** \| **Deprecated** \| **Index** \| **Help** \| **Weka's home** | |  |
| **PREV CLASS**   **NEXT CLASS** | **FRAMES**    **NO FRAMES**     **All Classes** |  |
| SUMMARY: NESTED \| FIELD \| CONSTR \| METHOD | DETAIL: FIELD \| CONSTR \| METHOD |  |


---
